# Psychometric properties of the Chinese version of the health behavior motivation scale: a translation and validation study

**DOI:** 10.3389/fpsyg.2024.1279816

**Published:** 2024-01-17

**Authors:** Yuanhui Ge, Chen Zheng, Xin Wang, Tao Liu

**Affiliations:** ^1^Department of Nursing, Jinzhou Medical University, Jinzhou, China; ^2^Nursing Department of Huaian Hospital of Huaian City, Huaian, China

**Keywords:** chronic diseases, pro-health behavior, health behavior motivation, psychometric properties, factor analysis

## Abstract

**Objective:**

This study’s objectives were to translate the Health Behavior Motivation Scale (HBMS) into Chinese and verify the scale’s validity and reliability among Chinese healthy adults.

**Method:**

The HBMS scales were translated into Chinese based on Brislin’s principles. The Chinese version of HBMS is created through translation, back translation, and cross-cultural adaptation. This investigation implemented the convenience sampling method to conduct a survey on 781 healthy respondents, utilizing the Chinese version of the HBMS and a general demographic questionnaire. We used AMOS (v28.0) and SPSS (v26.0) for statistical analysis. We employed test–retest reliability, split-half reliability, and internal consistency to assess the reliability of the translation questionnaire. Structure validity and content validity were used to assess validity.

**Results:**

The Chinese version of the Health Behavior Motivation Scale (HBMS) had a Cronbach’s alpha coefficient of 0.885, and the range of Cronbach’s alpha values for each dimension was 0.820–0.885. The scale’s test–retest reliability was 0.824, and its split-half reliability was 0.906. Five public factors with a cumulative variance contribution of 56.527% were retrieved from the exploratory factor analysis. Moreover, the factor loading value for each item exceeded 0.4.In confirmatory factor analysis, the indicators were reported as follows: χ^2^/df = 1.567, GFI = 0.900, CFI = 0.952, IFI = 0.952, TLI = 0.946, AGFI = 0.881, PGFI = 0.757, PNFI = 0.789, RMSEA = 0.039, and the results of the model fit metrics were within the reference range.

**Conclusion:**

The Chinese version of the HBMS exhibits strong discrimination, validity, and reliability. The tool effectively identifies the motivation of healthy people to engage in healthy behaviors. It can be used by healthcare practitioners to assist in the development of follow-up interventions to reduce the prevalence of chronic disease in older people and the incidence of chronic disease in populations of young and middle-aged people.

## 1 Introduction

The demographic makeup of China has altered as a result of the development of society and the transformation of the economic system, and the likelihood of an outbreak of numerous chronic diseases has increased. The burden of chronic diseases is rising, which has turned into a significant public health hazard to the country’s health when combined with the effects of the ecological environment, lifestyle modifications, and urban industrialization (Bernell and Howard, 2016). The prevalence of chronic illnesses like diabetes, dyslipidemia, and hypertension among the Chinese population is among the highest in the world. Studies have shown that the number of deaths due to chronic diseases is still at a high level. Globally, chronic diseases are already responsible for 41 million deaths by 2022, accounting for 74% of all deaths, with cardiovascular disease (CVD) being the leading cause of death from chronic diseases (Organization WH, 2023). In China, older adult people have a high prevalence of chronic diseases, and it is common for several illnesses to coexist. It is a significant issue influencing the health of the older adult because it is a nearly lifelong, irreversible disease. Long-term illness survival among the older adult population lowers the older adults quality of life, raises the risk of dependency and incapacity, and is not supportive of healthy aging. There are also many high-risk behaviors for chronic diseases, such as smoking, sitting down a lot, eating erratically, being overweight, and obesity, among Chinese young and middle-aged adults between the ages of 18 and 59. The number of young and middle-aged people with chronic diseases started to rise along with social pressure, which in turn raised the young and middle-aged population’s death rate and threatened their level of health (Neuhouser, 2019). It is clear that chronic disease incidence in China is on the rise and is becoming more common among younger people. Chronic disease is a condition with a protracted course and gradual progression that damages critical organ systems in the body. Long-term therapy increases both the need for care services and the associated medical costs. This would not only result in the diversion of resources related to medical and health services but also increase the cost of medical and health expenditures in China, which would have an impact on the long-term sustainability of the entire society and the entire nation. China currently needs to find solutions to two pressing issues: improving the health of the young and middle-aged population while reducing the prevalence of chronic diseases in this age group, and improving the management of chronic diseases in the older adults population while promoting successful aging.

Chronic diseases have a protracted incubation period, so prevention should be a major priority. Chronic illness risk is increased by poor health practices such as smoking, drinking, physical inactivity, a lack of exercise, and a poor diet (Ojo, 2019). According to research, we can successfully prevent and treat more than 80% of type 2 diabetes and cardiovascular disease by modifying individual behavior, and we can also lower the risk of cancer by at least 40% (Tian et al., 2023). The stage model of behavior change states that there are five different stages of achieving positive behavior change: pre-contemplation, contemplation, preparation, action, and maintenance (Coscioni et al., 2022; Mizuno et al., 2023). It involves eliciting certain motivations in order to encourage individuals to participate in an activity (Sujic et al., 2016; Sinelnikov and Wells, 2017). According to research, changes in healthy behaviors are driven by alterations in motivation. Measurement and assessment of people’s motivation for health behaviors is especially essential in light of the current state of chronic diseases in China. Health behavior motivations impact people’s likelihood of maintaining a healthy lifestyle as well as their openness to guidance from medical professionals (Michaelsen and Esch, 2021). Even though we are aware that altering health-related behaviors is crucial to preventing and managing chronic illness, we still lack a thorough understanding of the motivations underlying these modifications (Michaelsen and Esch, 2023). The majority of assessment instruments now in use focus on particular health behaviors, such as food or exercise (Bully et al., 2016; Ping et al., 2018; Guertin et al., 2020), but they often overlook the more universal motivations and attitudes that drive people to engage in these behaviors.

The Health Behavior Motivation Scale (HBMS), which comprises a total of 5 dimensions and 30 entries, was created in 2011 by Professor Magdalena Poraj-Weder and coworkers (Poraj-Weder et al., 2021). It has been demonstrated that this scale, which is used to measure healthy people’s motivation to undertake pro-health behaviors in Poland, has good psychometric qualities. Based on the theory of self-determination (Hricova et al., 2018; Migliorini et al., 2019), the scale separates intrinsic motivation and extrinsic motivation from aspects of autonomy, perception ability, relationships with others, and social environmental impact to understand the motivation process of people to implement health-related behaviors. Currently, the majority of health behavior assessment questionnaires are skewed toward particular topics (such as stress, physical activity, and diet) (Schmitter-Edgecombe et al., 2017; Lu et al., 2022) and can only quantify information as to the level of health behavior participation. The Treatment Self-Regulation Questionnaire (TSRQ), despite its wide usage, confines itself to merely four health behaviors, and the limited number of items within it may impact its reliability (Levesque et al., 2007). In order to investigate the motivations behind adopting pro-health habits, the HBMS scale qualitatively assesses information on pro-health behavioral activities. The scale assesses a wider range of areas than some questionnaires, focusing on more healthy adults and giving more thought to why people engage in pro-health behaviors. The scale’s validity and reliability have not been documented in pertinent studies, and it is now utilized relatively sparingly both domestically and internationally.

The measurement and evaluation of individuals’ motivation for health behaviors have become increasingly important, given societal advancements and rising health consciousness. However, reliable tools to measure these motivations are currently lacking in China. This study aimed to validate the psychometric properties of the HBMS, translate it into Chinese, cross-culturally debug it, and test the reliability and validity of the translated scale in healthy Chinese adults. This endeavor is expected to provide a crucial reference for promoting healthy aging throughout society.

## 2 Methods

### 2.1 Participants and research procedures

In China, this cross-sectional survey was carried out between April and July of 2023. Using a convenient sampling method, healthy adults were attracted from Liaoning Jinzhou and Shanxi Xinzhou to take part in the study. Prior to the survey starting, the team members had standardized training and acquired the usage of conventional language and expression techniques. The recreation center for residents was chosen as the location for the survey. Participants are required to conform to several conditions: (Bernell and Howard, 2016) the age of 18 years; (Organization WH, 2023) the absence of a non-communicable chronic condition; (Neuhouser, 2019) the absence of a conscious impairment; and (Ojo, 2019) voluntary involvement. People with systemic diseases, chronic non-communicable diseases (such as diabetes, hypertension, heart disease, etc.), genetic diseases, and psychiatric problems were excluded from the study to ensure its validity and reliability. Participants in the study were also not allowed if they were unable to communicate normally. The sample size needed for exploratory factor analysis and confirmatory factor analysis must be greater than the sample size for the minimal standard in order to assure their correctness. The final research comprised 781 healthy people in total.

### 2.2 Research tools

#### 2.2.1 General demographic characteristics questionnaire

Based on a literature review, the research team discussed and identified seven self-reported items, including gender, age, education level, place of residence, work status, marital status, and self-assessment of health status.

#### 2.2.2 Health behavior motivation scale (HBMS)

Prof. Magdalena Poraj-Weder and colleagues created the Health Behavior Motivation Scale (HBMS) in 2021 to assess the motivation of healthy individuals to engage in pro-health behaviors. It consists of 5 dimensions and 30 entries: Intrinsic regulation (6 entries), Integrated and identified regulation (6 entries), Introjected regulation (6 entries), External regulation (6 entries), and Non-regulation (6 entries). The scale is scored using the Likert 5 scale, with options including “DEFINITLY DISAGREE,” “MOSTLY DISAGREE,” “NEITHER AGREE NOR DISAGREE,” “MOSTLY AGREE,” and “DEFINITLY AGREE” corresponding to 1 to 5 points. The overall score ranges from 30 to 150, and the higher the score, the more consistent with the post-translation scale the statement about healthy adults’ “pro-health behavior motivation” is, as well as how obvious the incentive and maintenance mechanism for engaging in pro-health behaviors is.

### 2.3 Process

#### 2.3.1 Translation and cross-cultural adaptation of the scale

With Professor Magdalena Poraj-Weder’s permission, the scale was translated, and the Brislin translation model served as the foundation for the procedure. Two bilingual nursing graduate students who are familiar with the study background first independently translated the scale into Chinese, and then the research team debated and updated it to create the translated version. Two translators who were fluent in English and had never seen the original scale—one was a college English instructor and the other was a doctoral student—back-translated the translation scale. The backtranslated version was created following group debate and revision. The backtranslated version scale was cross-culturally debugged by psychologists in relevant domains using Chinese idiomatic use and Chinese cultural background. The translated scale’s readability and clarity were also evaluated by 30 healthy adults who were requested to participate in the pre-survey. Based on the preliminary analysis, the translated scale was modified to make its content clear and understandable, which resulted in the creation of the Chinese translation of the HBMS scale.

#### 2.3.2 Data collection

The group members visit both regions to seek out participants after training. At the beginning of the survey, the participants were identified and informed of the study’s objectives. To guarantee the confidentiality and privacy of this survey, the study procedure completely complies with the fundamental principles of medical research. The team member will review and retrieve the questionnaire when it has been completed on site. In the end, 781 questionnaires were collected, of which 47 were determined to be invalid and 734 to be valid. The questionnaires were successfully recovered in 93.98% of cases. 30 individuals were chosen to complete the questionnaire again after two weeks in order to evaluate the test–retest reliability of the Chinese version of the HBMS.

##### 2.3.2.1 Data analysis

SPSS 26.0 and AMOS 28.0 were used to conduct the statistical analysis. The mean and standard deviation are used to represent continuous data, whereas the percentage is used to explain categorical data. A statistically significant difference is shown by the value *p* < 0.05.

##### 2.3.2.2 Items analysis

The item analysis confirms the suitability and dependability of each input on the scale for the purpose of screening the entries (Gao et al., 2023). The first 27% (high score group) and the last 27% (low score group) of the total score can be determined by sorting the scale’s overall score from high to low. Determine whether there is a noticeable difference between the scores of each item in the two groups. *p* < 0.01 indicates a statistically significant difference between the high and low groups. Each item on the scale has a high degree of identification (critical ratio, CR > 3.0) (Yang et al., 2023). Determine the relationship between the item score and the scale’s overall score by calculating the correlation coefficient. *r* > 0.400 (Yang et al., 2021), which denotes a good correlation and homogeneity between each item and the overall score. Consider eliminating the item if, after removing each entry, Cronbach’s alpha value is higher than the post-translation scale.

##### 2.3.2.3 Reliability analysis

Reliability is an indicator for assessing the accuracy and consistency of measuring methods and relates to the stability and consistency of the survey scale (Zheng et al., 2023). Utilizing Cronbach’s alpha coefficient, split-half reliability, and test–retest reliability, the scale’s reliability was assessed (Mehta et al., 2018). For both the overall scale and the dimensions, Cronbach’s alpha coefficients were calculated; values greater than 0.700 (Leppink and Pérez-Fuster, 2017) denote a high degree of reliability and good internal consistency. The items on the scale were divided into two equal portions based on odd and even numbers using the parity-halving approach in order to evaluate the split-half reliability. The scores from the two sections are calculated, and the correlation coefficient is obtained. To determine the test–retest reliability of the results obtained from the two measurements, 30 participants were retested two weeks later.

##### 2.3.2.4 Validity analysis

Validity is an indication for assessing the accuracy of the scale and describes how closely the measurement instrument corresponds to the idea or phenomenon it is measuring (Dong et al., 2022). The validity of the scale is assessed using its content validity and structure validity. Content Validity Ratio (CVR): We invited ten experts from the respective fields to assess the necessity of scale items, categorizing them into three classes: ‘necessary’, ‘useful but unnecessary’, and ‘unnecessary’ (Almanasreh et al., 2019) Calculations were conducted using the CVR formula (Moradhaseli et al., 2020). According to Lawshe’s research, a CVR greater than 0.62 (Lawshe, 1975) is considered within the acceptable range. Content Validity Index (CVI): The Likert 4 scale was applied, with 1 denoting “no correlation” and 4 denoting “strong correlation.” The proportion of experts who rated “3 or 4” for each entry to the total number of experts is known as the content validity index of an item (I-CVI). The average of the I-CVI across all entries is known as the content validity index of the entire instrument (S-CVI). I-CVI > 0.78, S-CVI > 0.9, and higher values show good content validity (Merino-Soto and Livia-Segovia, 2022). Describe how the scale entries are a very good representation of the measurement content. Factor analysis was used to evaluate the scale’s structure validity, and exploratory factor analysis (EFA) was used to determine the scale’s potential factor structure. Confirmatory factor analysis (CFA) was used to verify the predetermined factor structure. For EFA and CFA, 734 samples were randomly split into two groups (n = 367). In the EFA, the Kaiser-Meyer-Olkin (KMO) value was above 0.6 (Kitayama et al., 2022), suggesting that the data is a positive definite matrix suited for factor analysis and that Bartlett’s test of sphericity passed the significance test threshold (*p* < 0.05). The principal component analysis (PCA) method was used for orthogonal rotation, and the following conditions had to be met: the eigenvalues >1, the total mutation interpretation rate of all common factor pairs reached more than 40%, and the factor load of each item was >0.4 (Yang et al., 2022). In CFA, the chi-square fitting goodness test method is used to perform the model fitness test. The model’s fitting index is as follows: the chi-square degree of freedom (χ^2^/df) ≤ 3; the root mean square error of approximation (RMSEA) ≤ 0.05; the goodness-of-fit index (GFI), the adjusted goodness-of-fit index (AGFI), the Tucker-Lewis index (TLI), the comparative fit index (CFI), and the incremental fit index (IFI) ≥ 0.9; the parsimonious goodness-of-fit index (PGFI) and the parsi-monious normed-of-fit index (PNFI) ≥ 0.5. This indicates that the model fits well and is acceptable (Barrett, 2007).

### 2.4 Ethics statement

Each participant in the survey was required to sign an informed consent form before participating, and they all have the right to withdraw from the study at any time. The confidentiality principle is upheld, and information privacy is ensured through this investigation. Jinzhou Medical University’s ethics review committee has given its approval for this project (JZMULL2023031).

## 3 Results

### 3.1 General information

A total of 734 valid questionnaires were collected in this study, with a recovery rate of 93.98%. Among them, 455 (62%) were females and 279 (38%) were males. 63.5% were young people aged 18–35 years, with a mean age of 35.34 ± 14.64 years. The majority (65.4%) considered their health to be “good.” Other demographic characteristics are shown in Table 1.

**Table 1:** General demography data (*n* = 734).

Factors	Group	*n*	%
Sex	Male	279	38.0
	Female	455	62.0
Age	18—35	466	63.5
	36—59	165	42.5
	≥60	103	14.0
Education level	Junior high school and below	57	7.8
	High school/secondary school	160	21.8
	Bachelor’s Degree and Above/College	517	70.4
Residence	Rural	263	35.8
	Urban	471	64.2
Work status	Employed	355	48.4
	Unemployed	379	51.6
Marital status	Unmarried/Divorced/Widowed	423	57.6
	Married	311	42.4
Health self-assessment	Poor	41	5.6
	Good	480	65.4
	Very good	213	29.0

### 3.2 Items analysis

According to the outcomes of the Critical Ratio approach, each item’s critical ratio (CR) is 7.630 ~ 16.726 (*p* < 0.001), and all are >3.0. The findings of the correlation study revealed a correlation coefficient of r = 0.381–0.571 (*p* < 0.01) between the item score and the overall scale score. As each item’s Cronbach’s alpha value after deletion, which ranged from 0.880 to 0.885, did not exceed the Cronbach’s alpha coefficient of the Chinese version of the scale, 30 items were retained as a consequence (Table 2).

**Table 2:** Item analysis of the Chinese version of the Health Behavior Motivation Scale.

Item	Critical ratio	Correlation coefficient between item and total score	Cronbach’s Alpha if item delete
1	7.630	0.399	0.883
2	9.297	0.410	0.883
3	8.896	0.414	0.883
4	9.761	0.439	0.883
5	11.176	0.473	0.882
6	9.954	0.415	0.883
7	11.260	0.486	0.882
8	14.684	0.547	0.880
9	11.789	0.474	0.882
10	13.579	0.512	0.881
11	14.810	0.541	0.881
12	13.791	0.524	0.881
13	14.597	0.543	0.881
14	11.934	0.473	0.882
15	17.208	0.571	0.880
16	15.260	0.541	0.881
17	11.730	0.484	0.882
18	13.551	0.526	0.881
19	13.115	0.492	0.882
20	16.395	0.517	0.881
21	15.594	0.516	0.881
22	15.142	0.539	0.881
23	15.653	0.530	0.881
24	14.980	0.518	0.881
25	16.726	0.508	0.882
26	15.193	0.489	0.882
27	13.582	0.441	0.884
28	13.787	0.441	0.884
29	13.395	0.431	0.884
30	10.753	0.381	0.885

### 3.3 Reliability analysis results

The Cronbach’s alpha coefficient for the Chinese version of the HBMS scale is 0.885, and the Cronbach’s alpha coefficient for each dimension is 0.820 to 0.894. The test–retest reliability is 0.824, and the split-half reliability is 0.906.

### 3.4 Validity analysis results

#### 3.4.1 Content validity

The correlation of entries on the Chinese HBMS scale was scored by seven experts in related domains. According to the findings, the range for the content validity index of an item (I-CVI) was from 0.800 to 1.000, and the content validity index for the entire instrument (S-CVI) came out to be 0.94. Moreover, the content validity ratio (CVR) was reported to vary within the range of 0.8 to 1.0.

#### 3.4.2 Exploratory factor analysis

**Figure 1:**
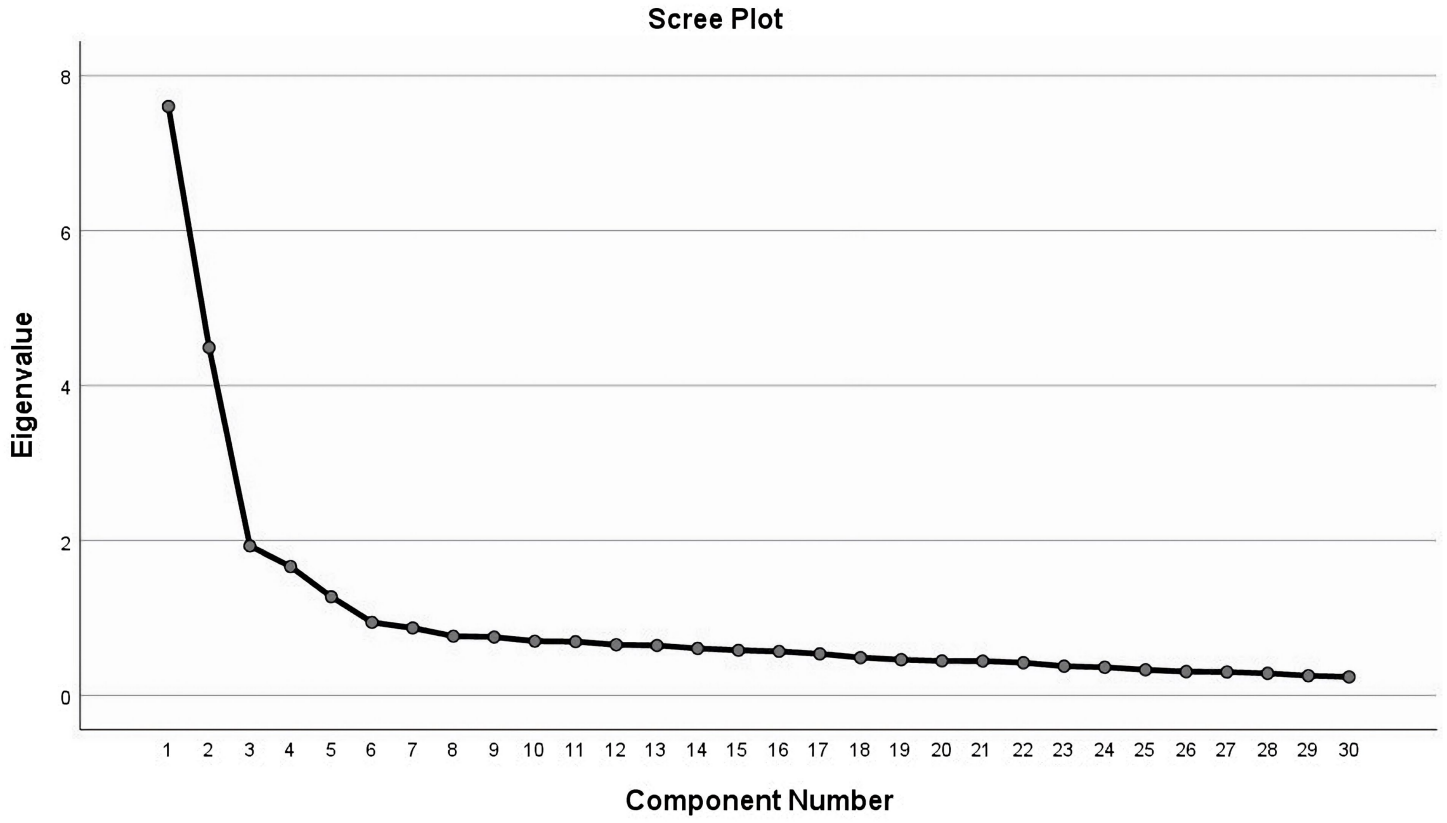
Exploratory factor analysis screen plot of the Chinese version of the Health Behavior Motivation Scale.

According to the results of the Bartlett spherical test (χ^2^ = 4658.664, df = 435, *p* <0.001), the KMO value was 0.895, the data satisfied the spherical hypothesis, and factor analysis was appropriate. The results of the screen plot were combined, further supporting the five-factor structural model (Figure 1). And the results revealed a cumulative explanatory variation of 56.527%. A total of 5 factors with eigenvalues >1 were extracted (Table 3). The factor load value is shown as 0.523–0.826 in Table 4.

**Table 3:** Initial Eigenvalue, percentage of variance and cumulative Percentage of Variance Explained.

Factors	Initial Eigenvalue	Percentage of Variance	Cumulative Percentage of Variance Explained
1	7.601	13.366	13.366
2	4.490	11.243	24.609
3	1.930	10.921	35.531
4	1.663	10.628	46.159
5	1.274	10.368	56.527

**Table 4:** Factor loadings from exploratory factor analysis of the Chinese version of the Health Behavior Motivation Scale.

Factor loading	Factor 1 (Intrinsic regulation)	Factor 2 (Integrated and identified regulation)	Factor 3 (Introjected regulation)	Factor 4 (External regulation)	Factor 5 [Non-regulation (Amotivation)]
1	0.591	-	-	-	-
2	0.608	-	-	-	-
3	0.714	-	-	-	-
4	0.693	-	-	-	-
5	0.690	-	-	-	-
6	0.652	-	-	-	-
7	-	0.556	-	-	-
8	-	0.656	-	-	-
9	-	0.736	-	-	-
10	-	0.692	-	-	-
11	-	0.619	-	-	-
12	-	0.637	-	-	-
13	-	-	0.656	-	-
14	-	-	0.723	-	-
15	-	-	0.675	-	-
16	-	-	0.659	-	-
17	-	-	0.698	-	-
18	-	-	0.696	-	-
19	-	-	-	0.523	-
20	-	-	-	0.782	-
21	-	-	-	0.798	-
22	-	-	-	0.724	-
23	-	-	-	0.600	-
24	-	-	-	0.609	-
25	-	-	-	-	0.780
26	-	-	-	-	0.826
27	-	-	-	-	0.817
28	-	-	-	-	0.817
29	-	-	-	-	0.782
30	-	-	-	-	0.724

#### 3.4.3 Confirmatory factor analysis

**Figure 2:**
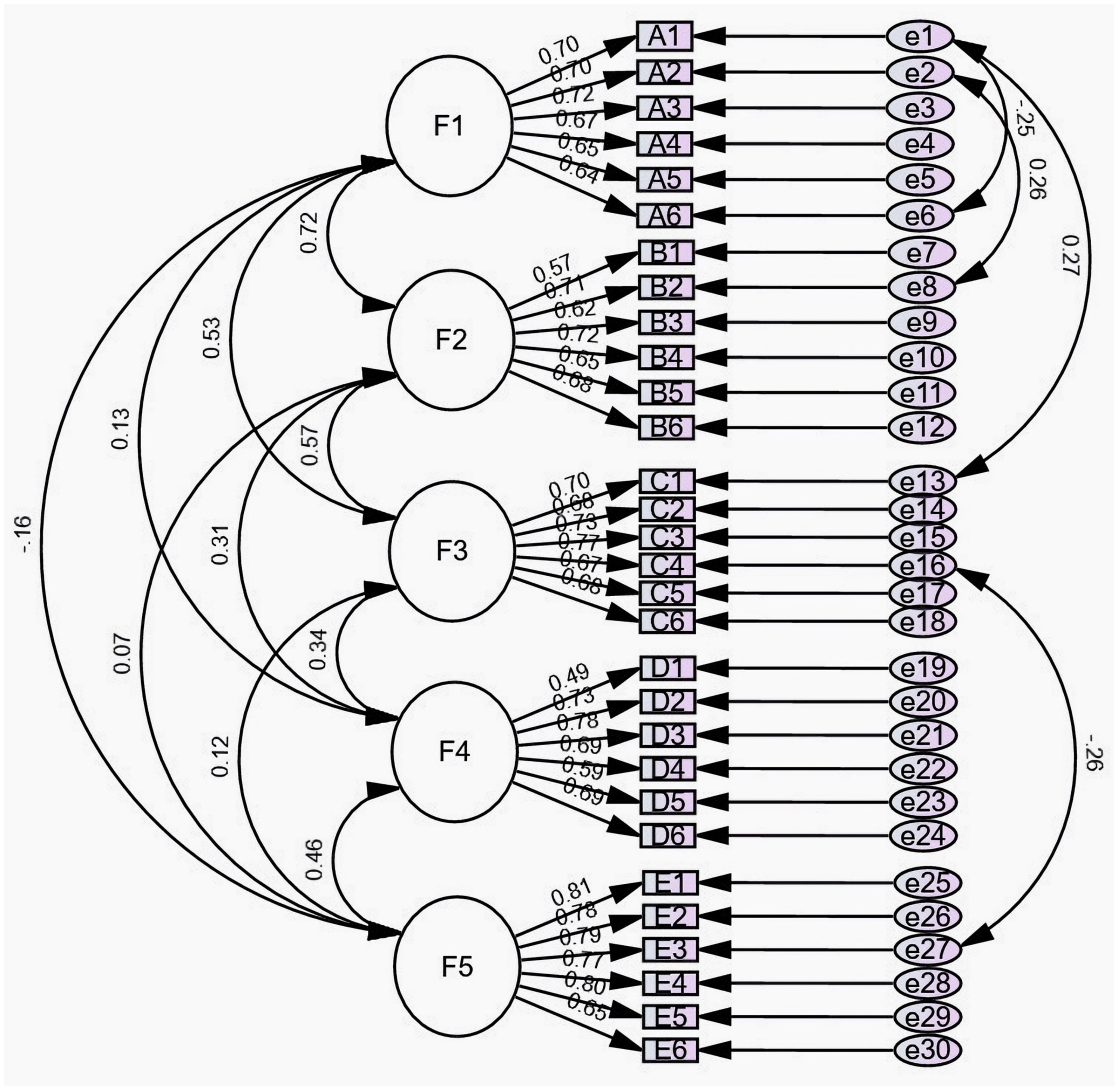
Standardized five-factor structural model of the Health Behavior Motivation Scale.

Based on the five-factor structural model, four residual paths were added according to the Modification Index (MI): e1 to e13, e1 to e6, e2 to e8, and e16 to e27. The fitting index of the revised model is: the chi-square/degree of freedom ratio (χ^2^/df) is 1.567, the Goodness-of-fit index (GFI) is 0.900, the Adjusted goodness-of-fit index (AGFI) is 0.881, the Parsimonious goodness-of-fit index (PGFI) is 0.757, the Incremental fit index (IFI) is 0.952, the Tucker-Lewis index (TLI) is 0.946, the Root Mean Square Error of Approximation (RMSEA) is 0.039, the Parsimonious normed fit index (PNFI) is 0.789, and the Comparative fitting index (CFI) is 0.952. All fitting indicators are within the range of reference values (Figure 2).

## 4 Discussion

### 4.1 The Chinese version of the HBMS scale has an ideal degree of distinction

The item analysis revealed strong homogeneity and good correlation between the scale’s items, as well as good discrimination (Zhang et al., 2022). The Cronbach’s alpha coefficient did not change beyond its initial value of 0.885 after each item was removed. Justify the retention of all items by stating that the Chinese version of the HBMS scale has a satisfactory identifying impact for each entry. To undertake cross-cultural debugging of the translated scale based on factors such as language expression habits, cultural background, and professional expertise, psychologists in related domains were recruited. Results from the pre-survey demonstrate that the scale’s layout makes it simple for participants of all ages to read and respond to questions. This scale is appropriate for assessing the motivation of Chinese adults in good health to engage in healthy behaviors. With higher scores on the scale than with lower scores, there is a persistent and more apparent motivation to undertake pro-health behaviors.

### 4.2 The Chinese version of the HBMS scale has good reliability

The degree of consistency of the measurement results is estimated by reliability (Yang et al., 2021). The dependability of the scale increases with the degree of uniformity of the measurement results. Both internal and external dependability were investigated as part of this study’s reliability assessment. Test–retest reliability serves as a proxy for extrinsic reliability, whereas Cronbach’s alpha coefficient and split-half reliability serve as proxies for intrinsic dependability. The reliability of the scale was good according to the results of this study, where the Cronbach’s alpha coefficient of the Chinese version of the HBMS scale was 0.885, the Cronbach’s alpha coefficient of each dimension was 0.820–0.894, and the reliability coefficient ranged from 0.800 to 0.900. 0.906 is split-half reliability. After two weeks, the test–retest reliability was 0.881. The higher the retest reliability and the more stable the scale, the closer this number is to 1. In conclusion, the Chinese translation of the HBMS scale is highly reliable.

### 4.3 The Chinese version of the HBMS scale has good validity

Validity is a measure of how accurate a scale measurement idea or phenomenon is estimated to be (Qi et al., 2022). The validity of the scale increases with the degree to which the anticipated research theme is reflected. The validity of this study was evaluated from two perspectives: structure validity and content validity. The degree to which a measurement tool captures the desired measurement notion is known as content validity. Structure validity defines and more fully comprehends the properties and capacities of the thing being measured and uses factor analysis to describe the underlying dimensions behind these factors. Content Validity Indices [I-CVI > 0.78 and S-CVI > 0.9 (Almanasreh et al., 2019)] and Content Validity Ratio (CVR), fluctuating between 0.8 and 1.0, all surpassed the standard reference of 0.62 (Lawshe, 1975), indicating robust content validity for the Chinese version of HBMS. The whole-screen plot results and the exploratory factor analysis demonstrate that the five-factor structural model that supports it is consistent with the original scale. Each factor corresponds to the same dimension as the original scale, and all factor loads are higher than 0.4 (Ye et al., 2018). The cumulative variation of the five common factors accounts for 56.527% of the overall variation. The confirmation factor analysis results demonstrated that the model’s fitting indicators were greater than the values used as the standard reference, and the model as built was suitable. In conclusion, the Chinese version of the HBMS scale has good validity.

### 4.4 Introductory significance

Chronic diseases have a significant negative impact on people’s quality of life in China and are now a significant public health issue that undermines the nation’s power to thrive economically and socially (Teixeira-Poit et al., 2019). Chronic diseases are becoming more prevalent in China, both in terms of incidence and population size. Therefore, early healthy lifestyle interventions are essential to encourage healthier behaviors, lower the incidence of chronic diseases, and postpone the initial appearance of a variety of health issues in healthy adults (Valanju et al., 2022). Having been subjected to linguistic and cultural adaptations, the Chinese variant of HBMS proves more fitting for the distinct situations and cultural milieu within the Chinese context and thus offers a more accurate depiction of the motivational landscape with respect to health behaviors among the Chinese population. The Chinese version of the HBMS includes important factors such as self-regulation ability, along with both internal and external drivers (Poraj-Weder et al., 2021). As a result, it can reveal the source of motivation for people to engage in healthy behaviors with greater accuracy. Moreover, compared to other similar types of scales, the evaluation results have higher practical applicability due to the pronounced differentiation and uniqueness inherent in the scale’s measuring indicators and how the motivational components are classified (Levesque et al., 2007; Bully et al., 2016; Ping et al., 2018; Guertin et al., 2020). Following its launch in China, this scale will offer a useful instrument to assess healthy adults’ motivation to engage in regular beneficial behaviors. Starting with an understanding of the motivational regulation of pro-health behaviors, healthcare professionals can effectively manage individual or population health risk factors, assist in maintaining and promoting the physical and mental health of Chinese residents, and provide them with health promotion intervention strategies. Future research should prioritize the incorporation of the Health Behavior and Motivation Scale (HBMS) into current health promotion and disease prevention approaches, along with customizing interventions for varied demographics (such as differing ages, genders, and socioeconomic statuses). Rigorous research remains necessary to discern the long-lasting impacts of the HBMS and its potential in aiding individuals to modify their health-related behaviors sustainably.

## 5 Limitation

Some of the issues that arose while doing this study merit discussion and consideration. In this study, a measurement tool was used to conduct the self-assessment. Subjective considerations were taken into account when identifying the traits of the subjects, and a deviation was unavoidable. In light of geographical and cultural variations, there are disparities in public perceptions of health behavior. Thus, extrapolating from the data gleaned from two provinces to represent other regions inevitably introduces potential limitations. Subsequent research endeavors have to span several locations and areas in order to enhance the precision of the data while comprehensively accounting for all variables that could impact the study’s outcomes.

## 6 Conclusion

When translated and validated, the Health Behavior Motivation Scale (HBMS) is a valid and reliable measurement tool for assessing healthy people’s motivation to adopt healthy behaviors and clarifying the motive. This research actively aligns with, and supports, the objectives of China’s “Healthy China Program.” The ultimate goal of our study was to contribute to national health enhancement and the establishment of a healthier China.

## Data availability statement

The original contributions presented in the study are included in the article/Supplementary material, further inquiries can be directed to the corresponding author.

## Ethics statement

The studies involving humans were approved by Ethics Review Committee of Jinzhou Medical University (JZMULL2023031). The studies were conducted in accordance with the local legislation and institutional requirements. The participants provided their written informed consent to participate in this study.

## Author contributions

YG: Data curation, Software, Writing – original draft, Writing – review & editing. CZ: Conceptualization, Supervision, Writing – review & editing. XW: Investigation, Writing – review & editing. TL: Conceptualization, Methodology, Writing – review & editing.

## Supplementary material

SUPPLEMENTARY TABLE 1 Raw data for the Chinese version of the health behavior motivation scale.

SUPPLEMENTARY TABLE 2 Original questionnaire for the Chinese version of the health behavior motivation scale.

SUPPLEMENTARY DATA SHEET 1 SPSS database for the Chinese version of the health behavior motivation scale.
