# Supplementary material for: Psychometric properties of the Chinese version of the health behavior motivation scale: a translation and validation study
Source: Front Psychol. 2024 Jan 17;15:1279816. doi: 10.3389/fpsyg.2024.1279816 (PMC10827909; doi:10.3389/fpsyg.2024.1279816)
Supplement: SUPPLEMENTARY TABLE 2 — Original questionnaire for the Chinese version of the health behavior motivation scale. [file Table_2.DOCX]

**Research on Healthy Adults Adopting Healthy Behaviors**

Hello! You are participating in a topic study that aims to explore the “Motivation of Healthy Adults to Health Behavior” survey, which can significantly improve health promotion and serve as a foundation for future health interventions. We sincerely hope you will carefully read the following questions and provide your thoughtful responses because your responses are crucial to us. No names are necessary; the survey data will be uploaded anonymously. Thank you for your assistance!

Please fill in some of your basic information

1.Sex

| ○Male |
| --- |
| ○Female |

2. Age

| ○18~35 |
| --- |
| ○36~59 |
| ○≥60 |

3. Your age is [fill in the blank]

_________________________________

4. Education level

| ○Junior high school and below |
| --- |
| ○High school/secondary school |
| ○Bachelor's Degree and Above/College |

5. Residence

| ○rural |
| --- |
| ○urban |

6. Work status

| ○Employed |
| --- |
| ○Unemployed |

7. Marital status

| ○Unmarried/Divorced/Widowed |
| --- |
| ○Married |

8. Health self-assessment

| ○Poor |
| --- |
| ○Good |
| ○ Very good |

HEALTH BEHAVIOR MOTIVATION SCALE (HBMS)

   This questionnaire contains statements reflecting various reasons why people engage in pro-healthy behaviors. The HBMS refers to a category of health behaviors which is understood as personal routine daily health activities, named health practices. Please, read each of these statements carefully and, by putting an X in an appropriate column, indicate how well this statement describes YOUR reasons for undertaking pro-healthy behaviors.
   There is no right or wrong answer. Any answer you give is good if only it is true, that is, in accordance with what you think and feel.

Why do you undertake pro-health behaviors?

| Dimension 1:Intrinsic regulation | DEFINITLY DISAGREE | MOSTLY DISAGREE | NEITHER AGREE NOR DISAGREE | MOSTLY AGREE | DEFINITLY AGREE |
| --- | --- | --- | --- | --- | --- |
| Because it makes me happy | 1 | 2 | 3 | 4 | 5 |
| Because it gives me vitality | 1 | 2 | 3 | 4 | 5 |
| Because it gives me vigor | 1 | 2 | 3 | 4 | 5 |
| Because it gives me pleasure | 1 | 2 | 3 | 4 | 5 |
| Because it’s a lot of fun | 1 | 2 | 3 | 4 | 5 |
| Because it drives me to act | 1 | 2 | 3 | 4 | 5 |

| Dimension 2:Integrated and identified regulation | DEFINITLY DISAGREE | MOSTLY DISAGREE | NEITHER AGREE NOR DISAGREE | MOSTLY AGREE | DEFINITLY AGREE |
| --- | --- | --- | --- | --- | --- |
| Because I treat it as an important and ongoing task to undertake | 1 | 2 | 3 | 4 | 5 |
| Because it’s my life plan | 1 | 2 | 3 | 4 | 5 |
| Because it is my current life choice | 1 | 2 | 3 | 4 | 5 |
| Because it is an essential part of my life | 1 | 2 | 3 | 4 | 5 |
| Because the “here and now” is important for me | 1 | 2 | 3 | 4 | 5 |
| Because it is congruent with my currently set life goals | 1 | 2 | 3 | 4 | 5 |

| Dimension 3:Introjected regulation | DEFINITLY DISAGREE | MOSTLY DISAGREE | NEITHER AGREE NOR DISAGREE | MOSTLY AGREE | DEFINITLY AGREE |
| --- | --- | --- | --- | --- | --- |
| Because whenever I neglect my health I feel guilty | 1 | 2 | 3 | 4 | 5 |
| Because I feel remorse when I neglect my health | 1 | 2 | 3 | 4 | 5 |
| Because I feel remorse when my health becomes less of a priority | 1 | 2 | 3 | 4 | 5 |
| Because I feel guilty when I stop taking care of my health | 1 | 2 | 3 | 4 | 5 |
| Because I feel remorse if I don’t take care of my health | 1 | 2 | 3 | 4 | 5 |
| Because if I don’t take care of my health I feel like I’m acting wrong | 1 | 2 | 3 | 4 | 5 |

| Dimension 4:External regulation | DEFINITLY DISAGREE | MOSTLY DISAGREE | NEITHER AGREE NOR DISAGREE | MOSTLY AGREE | DEFINITLY AGREE |
| --- | --- | --- | --- | --- | --- |
| Because others expect me to take care of my health | 1 | 2 | 3 | 4 | 5 |
| Because I want to meet others’ expectations | 1 | 2 | 3 | 4 | 5 |
| Because I want to make others happy | 1 | 2 | 3 | 4 | 5 |
| Because I don’t want to disappoint people around me | 1 | 2 | 3 | 4 | 5 |
| Because I feel pressure from my social environment | 1 | 2 | 3 | 4 | 5 |
| Because I don’t want others to nitpick my action | 1 | 2 | 3 | 4 | 5 |

| Dimension 5:Non-regulation | DEFINITLY DISAGREE | MOSTLY DISAGREE | NEITHER AGREE NOR DISAGREE | MOSTLY AGREE | DEFINITLY AGREE |
| --- | --- | --- | --- | --- | --- |
| I don’t do it because it causes me to feel lost | 1 | 2 | 3 | 4 | 5 |
| I don’t do it because a feeling of helplessness arises in me | 1 | 2 | 3 | 4 | 5 |
| I don’t do it because I could fail again | 1 | 2 | 3 | 4 | 5 |
| I don’t do it because it's beyond me | 1 | 2 | 3 | 4 | 5 |
| I don’t do it because I feel like I can’t | 1 | 2 | 3 | 4 | 5 |
| I don’t do it because it's beyond me | 1 | 2 | 3 | 4 | 5 |

Thank you for your cooperation!
